# Maternal Under- and Over-Nutrition during Gestation Causes Islet Hypertrophy and Sex-Specific Changes to Pancreas DNA Methylation in Fetal Sheep

**DOI:** 10.3390/ani11092531

**Published:** 2021-08-28

**Authors:** Maria Peterson, Mary Gauvin, Sambhu Pillai, Amanda Jones, Katelyn McFadden, Katelynn Cameron, Sarah Reed, Steven Zinn, Kristen Govoni

**Affiliations:** 1 Department of Fisheries, Animal and Veterinary Science, University of Rhode Island, Kingston, RI 02891, USA; 2 Department of Animal Science, University of Connecticut, Storrs, CT 06289, USA; marygauvin18@gmail.com (M.G.); sambhuvet@gmail.com (S.P.); amanda_2.jones@boehringer-ingelheim.com (A.J.); katelyn.mcfadden2@gmail.com (K.M.); katelynncameron22@gmail.com (K.C.); sarah.reed@uconn.edu (S.R.); steven.zinn@uconn.edu (S.Z.); kristen.govoni@uconn.edu (K.G.)

**Keywords:** beta cell function, fetal programming, DNA methylation, endocrine pancreas, sheep

## Abstract

**Simple Summary:**

The development of the fetal pancreas tissue can be affected during gestation by alterations to the intrauterine environment, often referred to as fetal programming. However, the mechanisms by which fetal programming predisposes offspring to reduced β-cell function later in life are poorly understood. The aims of this study were to (1) determine how under or over-nutrition during gestation can affect the growth and development of the pancreas tissue during gestation and (2) determine how the DNA methylation patterns of the pancreas tissue could be affected. We were able to determine that maternal under- and over-nutrition during gestation altered offspring pancreas structure causing reductions in islet size and number. Additionally, the changes in DNA methylation patterns were determined to be in a diet-specific and sex-dependent manner. These data are being used to better understand the mechanisms by which the development of the pancreas is affected by fetal programming with the ultimate goals of developing appropriate intervention strategies for these offspring.

**Abstract:**

The mechanisms by which fetal programming predisposes offspring to reduced β-cell function later in life are poorly understood. We hypothesized that maternal under- and over-nutrition during gestation would negatively affect offspring pancreas development and alter DNA methylation patterns. Pregnant ewes (*n* = 78) were fed 100, 60, or 140% of NRC requirements beginning at d 30.2 ± 0.2 of gestation. The fetuses are referred to as CON, RES, and OVER, respectively. Fetal pancreas tissue was collected at d 90 or 135 of gestation or within 24 h of birth. Tissue was preserved for histological (*n* = 8 to 9 offspring per treatment per time point) and DNA methylation analyses (*n* = 3 to 4 fetuses per treatment per sex). At d 135, OVER exhibited an increased islet size, reduced islet number, and greater insulin positive area compared with CON (*p* ≤ 0.03). An increased islet size was also observed at d 135 in RES (*p* ≤ 0.03) compared with CON. Cellular proliferation was reduced at birth in OVER vs. CON (*p* = 0.01). In the RES vs. CON females, 62% of the differentially methylated regions (DMRs) were hypomethylated (*p* ≤ 0.001). In the RES vs. CON males, 93% of the DMRs were hypermethylated (*p* ≤ 0.001). In OVER, 66 and 80% of the DMRs were hypermethylated in the female and male offspring compared with CON (*p* ≤ 0.001). In conclusion, changes to maternal diet during pregnancy affects the islet hypertrophy and cellular proliferation of the offspring at early post-natal time points. Additionally, changes in DNA methylation patterns appear to be in a diet-specific and sex-dependent manner.

## 1. Introduction

The pancreas has a vital role in regulating blood glucose homeostasis and digestion [1]. Consequently, alterations to pancreas tissue structure and function can have detrimental effects on the health of the individual. During embryonic development, the establishment of the pancreatic bud from mesenchymal stem cells occurs at d 9.5, 24, and 26 of gestation in mice, sheep, and humans, respectively [2,3]. Embryonic pancreatic development involves the expression of several key transcription factors to establish the endocrine cellular lineages (α, β, δ, and γ) as well as differentiate the acinar tissue [4]. Specifically, the expression of factors such as promoter factor (*PDX*) *1*, *Nerogenin (NEUROG)3*, and insulin gene enhancer protein (*Isl)1* are essential to the differentiation of α, β, and δ cells, which will form and coalesce into the islets of Langerhans [4]. The expression of *PDX1* also has an integral role in the maintenance of β cell mass postnatally [4]. Therefore, an altered expression of these factors in the developing pancreas could have detrimental effects on the health and function of the pancreas tissue during prenatal development and postnatal life. The development of the fetal pancreas tissue can be affected during gestation by alterations to the intrauterine environment, often referred to as fetal programming [5].

Changes to the intrauterine environment can be caused by many factors (e.g., disease, stress, placental insufficiency, under-nutrition. This, in turn, causes an adaptive response in the fetus leading to alterations in organ development in the offspring [6]. Given that nutrient restriction occurs in developing countries and over-nutrition in westernized countries [7], understanding the effects of maternal diet on the development of pancreatic tissue is needed. Epidemiological studies have linked fetal exposure to nutrient restriction during pregnancy to diabetes later in life [8]. Furthermore, primary research conducted in sheep and rats has demonstrated that maternal nutrient restriction or over-nutrition during pregnancy impacts the circulating insulin concentrations in mature offspring [9] and affects β-cell mass [10,11], with these effects being permanent and multigenerational [11]. How maternal diet affects circulating insulin secretion appears to be diet specific and dependent on when the fetus is exposed to the alterations in maternal diet. However, the mechanisms by which maternal nutrition affects the fetal growth and development of the pancreas are largely unknown. Consequently, this limits the knowledge of how pancreatic function may be altered in postnatal life.

One potential mechanism for the regulation of the development of the pancreas is DNA methylation. This epigenetic modification occurs at CpG dinucleotides where the cytosines are methylated by the corresponding methyltransferases [12]. DNA methylation regulates the availability of a given DNA region for transcription. Specifically, hypermethylation results in a reduction in transcription, whereas hypomethylation will result in the increased availability of the DNA for transcription [12]. Changes to DNA methylation have been identified as a result of maternal diet in human and rodent models in several different tissue types [13,14,15]. However, there is limited information on the effects of maternal nutrition during gestation on the DNA methylation patterns of the offspring pancreas tissue.

The objective of this study was to identify how maternal restricted- and over-nutrition during gestation affect pancreas DNA methylation patterns and pancreas development using an ovine model. We hypothesized that under- and over-feeding ewes would (1) reduce the islet size and number in the fetal pancreas, (2) alter the hormone positive area and architecture of the fetal pancreas, and (3) reduce the DNA methylation of genes involved in fetal pancreas development. To test these hypotheses, we evaluated the fetal pancreas at mid- and late gestation, as well as within 24 h of birth after exposure to poor maternal nutrition.

## 2. Materials and Methods

### 2.1. Animals

All animal procedures were reviewed and approved by the University of Connecticut Institutional Animal Care and Use Committee (A13-059). Experimental design, animal care, and sample collection were previously described [16] but will be described briefly herein. Multi-parous western whiteface ewes (*n* = 82; approximately ≥2 years of age). Ewes were estrus synchronized and bred to one of four related Dorset rams. Ultrasound was subsequently used to confirm pregnancy. Ewes were individually housed starting at 31 ± 0.2 d of gestation and fed a diet that was either 100% (*n* = 27), 60% (*n* = 28), or 140% (*n* = 27) of NRC requirements for a ewe pregnant with twins [17]. Ewe body weights and body condition scores were similar prior to the start of the study. Four ewes were excluded due to reasons unrelated to the study. Offspring from these ewes are referred to as CON, RES, and OVER, respectively. A subset of ewes (*n* = 5–7 per treatment per time point; [16]) were euthanized at d 90 or 135 of gestation and fetal tissues were collected. Another subset of ewes gave birth, at which point the lambs were euthanized within 24 h of parturition to comprise the birth time point. Pancreas tissue (50–500 g depending on pancreas size) was excised from each fetus (*n* = 10–13 fetuses). Tail portions of the pancreas tissue were frozen in optimal cutting temperature medium (Thermofisher, Waltham, MA, USA) over liquid nitrogen cooled isopentane for later histological analyses. Additional pieces of whole pancreas tissue from the tail were snap frozen in liquid nitrogen and stored at −80 °C.

### 2.2. Histological Analyses

Samples collected from fetuses at d 90 (*n* = 8 fetuses per treatment; 3–5 females per treatment; 4–6 males per treatment) and 135 (*n* = 9 fetuses per treatment; 3–7 females per treatment; 3–6 males per treatment) of gestation and at birth (*n* = 8 live newborn lambs per treatment; 6–7 females per treatment; 1–2 males per treatment) were used for histological analyses. Antibodies used included insulin (1:500, Dako, Santa Clara, CA, USA), somatostatin (1:500, Dako), glucagon (1:500; Sigma Aldrich; St. Louis, MO, USA), and phosphohistone-H3 (PHH3; 1:125; Millipore, Burlington, MA, USA). Protocols were adapted from Limesand et al. ([18]). For all histological analyses, 3–4 sections of pancreas tissue per animal were used. To evaluate β-cell proliferation, sections were co-stained with insulin and PHH3. Sections were also co-stained with glucagon and somatostatin. The tail portion of the pancreas was sectioned at −20 °C at a thickness of 5 µm. Sections were warmed on a hot plate to adhere sections to the slide and fixed using a 4% solution of paraformaldehyde (Thermofisher, Waltham, MA, USA). Sections were then washed using a gradient of Triton-X in Tris buffered saline (TBS) solution (once with 0.25% Triton-X, then twice using 0.025% Triton-X) for 5 min. For immunostaining, antigen retrieval was performed using sodium citrate and citric acid-based buffers at the 60% power setting of a microwave for 10 min in a pressure cooker. For insulin/PHH3 analyses, sections were digested for 30 s in 10 µg/mL proteinase K in 10 mM Tris-HCL (Thermofisher) and immediately rinsed with TBS. Immunostaining was visualized using Alexafluor 596 and 488 secondary antibodies (1:500; Invitrogen, Carlsbad, CA, USA). All images were taken at 40x magnification. Nuclei were visualized using DAPI (Abcam, Cambridge, MA, USA). Cellular apoptosis was determined using a TUNEL cell apoptosis assay by Roche (Sigma Aldrich). Sections were imaged using a Zeiss AxioObserver microscope. Image false coloring and quantification were performed in ImageJ (NIH, Bethesda, MD, USA). For insulin, glucagon, and somatostatin staining, the total positive area was quantified. For PHH3, the number of positive nuclei were enumerated. When quantifying PHH3 positive cells, only those animals with PHH3 positive cells were utilized (*n* = 5–7 per treatment per time point). Therefore, proliferating β-cells were considered those that were PHH3 positive/Insulin. When applicable, total positive area was normalized using the weight of the pancreas [18]. The percent of cells undergoing apoptosis was determined by enumerating the number of TUNEL-positive cells and dividing that by the total number of nuclei within a field of view. To measure islet size and number, sections were stained using hematoxylin and eosin Y (Sigma Aldrich using a standard protocol. Islet size and number was quantified using ImageJ. Specifically, individual islets in a field of view were counted. Islet size was determined by drawing around the outside of each islet with the freehand tool in ImageJ and quantifying this area using the software.

### 2.3. DNA Isolation and Sequencing

For the DNA methylation analyses, pancreas tissue from d 135 fetuses (3–4 fetuses per sex per treatment) were utilized. This time point was selected based on the even sex distribution, which allowed us to perform the appropriate comparisons. Pancreas DNA was isolated using a QIamp Fast DNA isolation kit (Qiagen, Valencia, CA, USA) and bisulfite treated using a commercially available kit (Zymo; Irvine, CA, USA). Libraries were prepared at the University of Connecticut Center for Genomic Innovation using Illumina Tru-seq adaptors (San Diego, CA, USA) and sequenced using an Illumina Miseq.

### 2.4. Data Analyses

Pancreas weights were analyzed as a percent of BW. Pancreas weight data are presented in whole gram unadjusted amounts. Fixed effects and interactions were utilized as previously described in [16]. For histological analyses, data were analyzed using PROC MIXED in SAS (Cary, NC, USA). A very limited number of male offspring were born at the birth time point and, therefore, sex was excluded from the model for the aforementioned analyses. When performing our histological analyses, twin offspring were used whenever possible. If a singleton or triplet lamb had to be used to increase the *n* per treatment, then offspring from similar litter sizes were included across the other treatment groups. In some cases, there were no singleton or triplet pregnancies within a given treatment group and, therefore, could not be included. Specifically at d 90, 7 twins and 1 singleton lamb were used for CON; 6 twins, 1 singleton, and 1 triplet lamb were used for RES; and 6 twins, 1 singleton, and 1 triplet were used for OVER. At d 135, 6 twins, 2 triplets, and 1 singleton lamb were used for CON; 7 twins and 2 triplet lambs were used for RES; and 8 twins and 1 singleton were used for OVER. At birth, 6 twins and 2 triplets were used for CON, 8 twins were used for RES, and 8 twins were used for OVER. If triplets were used, the larger triplet offspring were utilized in an effort to exclude the undersized member of the trio who might have experienced additional programming effects. Due to the low number of singleton and triplet pregnancies in this study, litter size was not included in the model due to low statistical power. As a result the analyses could not be completed with this variable included. Data were considered different from control when *p* ≤ 0.05 and tendencies when *p* ≤ 0.1 and >0.05. Fixed effects included maternal diet and stage of gestation. When selecting animals for RRBS, preference was given for twin offspring. For DNA methylation analyses, resulting reads were trimmed and filtered (*q* score ≥ 30) using TrimGalore and mapped to a bisulfite converted *Ovis aries* reference annotation (Oar_V.3.1) using Bismark. Differentially methylated loci (DML) and regions (DMRs) were determined using the dispersion shrinkage for sequencing analysis package (DSS) in R studio. Loci were considered differentially methylated when *q* ≤ 0.01 and delta = 0.10. Regions were considered differentially methylated when *p* ≤ 0.001 and delta = 0.10. A DMR was considered to be located in a prospective promoter/enhancer region when it was ≤5 kb upstream from a gene. Typically, promoter and enhancer regions are located −40 bp–1.5 kbp away from the translational start site; however, these regions can be larger and need to be experimentally determined [19,20]. Therefore, in the interest of not excluding potentially relevant information, this region was expanded to 5 kbp. Functional annotations for DMRs were performed using DAVID v 6.8 [21,22]. Data files from this study are being added to an appropriate repository and the corresponding data file identifiers will be added prior to publication.

## 3. Results

### 3.1. Fetal Islet Size and Number

**Figure 1:**
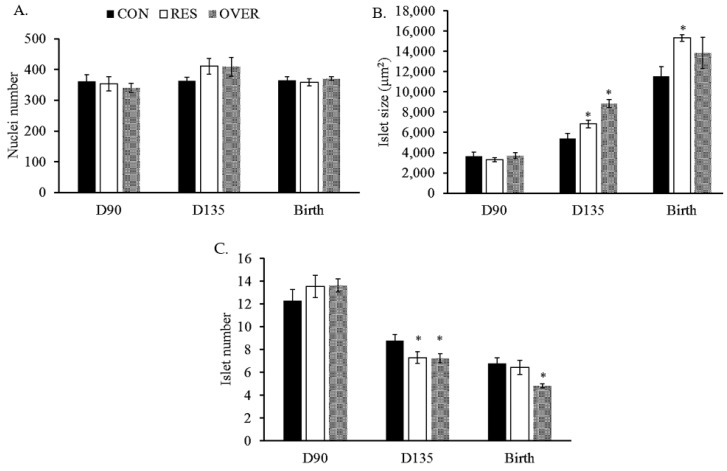
Alterations to maternal diet increase fetal pancreas islet size and reduces islet number. Nuclei number (**A**), Islet size (**B**) and number (**C**) were quantified using ImageJ. Pancreas islets from fetuses were stained with hematoxylin and eosin and imaged at 40× magnification. * *p* ≤ 0.05 compared with CON.

No difference in fetal pancreas weights was observed at d 90 (CON: 0.69 ± 0.04 g; RES: 0.55 ± 0.04 g; OVER: 0.70 ± 0.08 g) and d 135 (CON: 4.02 ± 0.30 g; RES: 3.15 ± 0.30 g; OVER: 4.08 ± 0.20 g) of gestation or at the birth (CON: 4.11 ± 0.33 g; RES: 4.19 ± 0.29 g; OVER: 3.83 ± 0.35 g) time points (*p* ≥ 0.11). No difference was observed in the pancreas tissue nuclei number at any of the three time-points measured (*p* ≥ 0.38; Figure 1A).

At d 135 of gestation, the islet size (Figure 1B) was 26 and 63% greater in the RES (6830 ± 388 μm^2^; *p* = 0.01) and the OVER (8836 ± 299 μm^2^: *p* < 0.01) offspring compared with CON (5404 ± 477 μm^2^). At d 135 of gestation, the islet number was reduced by 17% in RES (7.2 ± 0.52; *p* = 0.02) and OVER (7.2 ± 0.39; *p* = 0.04; Figure 1C) vs. CON (8.8 ± 0.53). At birth, the islet number was reduced in the OVER lambs (4.8 ± 0.17; *p* = 0.01) by 28% vs. CON (6.7 ± 0.50). Additionally, the islet size was 32% greater in RES at birth (*p* < 0.05; 15,326 ± 388 μm^2^) compared with CON (11,553 ± 952 μm^2^). No differences in the islet size or number were observed in the pancreas tissue of the day-90 fetuses (*p* ≥ 0.46).

### 3.2. β-Cell Proliferation and Apoptosis

**Figure 2:**
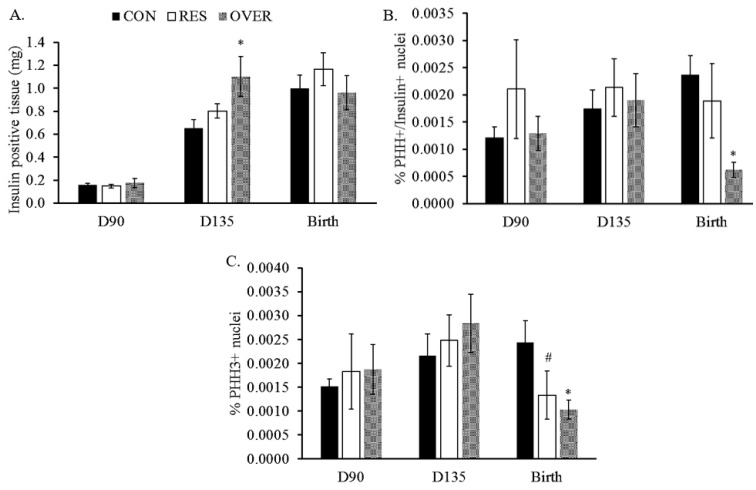
Maternal over-nutrition increases insulin positive area in the pancreas of fetuses at d 135 of gestation and reduces cellular proliferation at birth. Insulin positive area (**A**), percent PHH3/Insulin positive nuclei (**B**) and PHH3 positive nuclei (**C**) were quantified and false colored using ImageJ. Data were normalized using the mass of the pancreas or total number of nuclei where applicable. * *p* ≤ 0.05, ^#^ 0.05 < *p* ≤ 0.10 compared with CON.

At d 135 of gestation, the insulin positive area was 69% greater in OVER (1.10 ± 0.17 μm^2^/g; *p* = 0.01; Figure 2A vs. CON fetuses (0.65 ± 0.07 μm^2^/g) and tended to be 37% greater than the RES fetuses (0.80 ± 0.06 μm^2^/g; *p* = 0.07).

At birth, the β-cell proliferation was reduced by 73% in the OVER lambs (2.3 × 10^−3^ ± 3.0 × 10^−4^%) compared with the CON lambs (6.2 × 10^−4^ ± 1.4 × 10^−4^%; *p* = 0.01; Figure 2B) at birth. Likewise, there were 45 and 57% fewer total PHH3 positive cells in the RES (1.3 × 10^−3^ ± 5.0 × 10^−4^%; *p* = 0.07; Figure 2C) and OVER (1.0 × 10^−3^ ± 1.0 × 10^−4^%; *p* = 0.01) lambs, respectively, vs. the CON (2.4 × 10^−3^ ± 4.4 × 10^−4^%) lambs. Based on the reduction in cellular proliferation observed at birth, a TUNEL assay was performed to determine if there was an increased rate of apoptosis in the pancreas tissue. No difference in apoptosis was observed in the pancreas tissue of the offspring (1.51 ± 0.38%; 1.03 ± 0.41%; and 1.13 ± 0.44% for the CON, RES, and OVER fetuses, respectively; *p* = 0.67). No differences in the total cellular proliferation or proliferating β-cells were observed at d 90 or d 135 of gestation (Figure 2C; *p* ≥ 0.46).

### 3.3. Glucose and Somatostatin Positive Area

**Figure 3:**
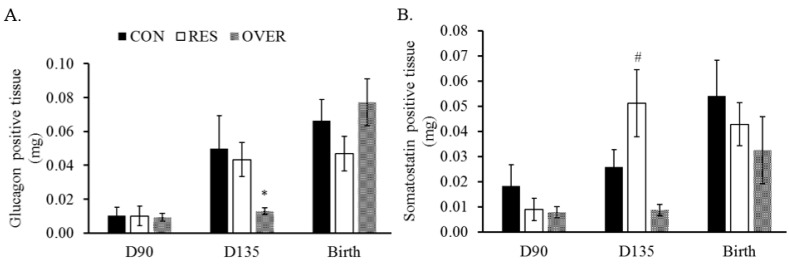
Maternal over-nutrition reduces glucagon positive area and maternal under-nutrition increases somatostatin positive tissue in the pancreas of offspring. Glucagon positive (**A**) and somatostatin positive tissue (**B**) were quantified, and images were false colored using ImageJ. Data were normalized using pancreas mass. * *p* ≤ 0.05, ^#^ 0.05 < *p* ≤ 0.10 compared with CON.

The glucagon positive area was 74% less in OVER (0.01 ± 0.002 μm^2^/g) vs. CON (0.05 ± 0.01 μm^2^/g; *p* = 0.05; Figure 3A) at d 135 of gestation.

The somatostatin positive area was greater in RES (0.05 ± 0.01 μm^2^/g; *p* = 0.06) at d 135 of gestation compared with the CON (0.02 ± 0.006 μm^2^/g) and OVER fetuses (0.008 ± 0.002 μm^2^/g; *p* < 0.01; Figure 3B). No differences in the glucagon or somatostatin positive tissue were observed at d 90 of gestation or at the birth time point (*p* ≥ 0.40; Figure 3A,B).

### 3.4. Sequencing Information

The male and female offspring were separated by sex for analyses. The sequencing of samples from male offspring yielded, on average, 5,030,545 reads (Table 1).

Likewise, the samples from female offspring, on average, produced 6,029,673 raw reads. Across all the treatment groups, 23% (male offspring) and 42% (female offspring) of reads mapped back to the reference annotation post trimming (Table 1).

### 3.5. Differentally Methylated Loci (DML) and Regions (DMRs)

**Figure 4:**
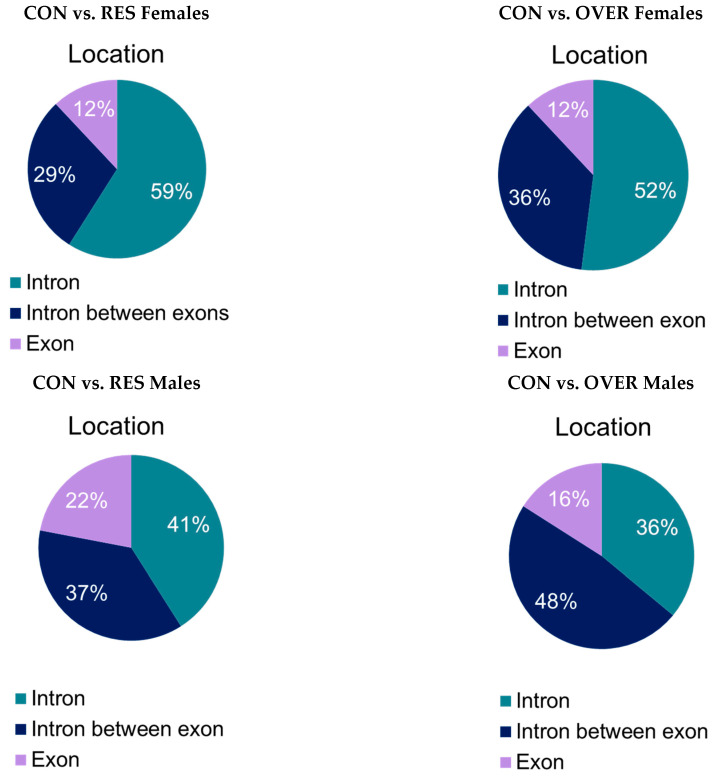
Differentially methylated region (DMR) locations. A total number of 34, 64, 48, and 25 DMRs were identified for CON vs. RES females, CON vs. RES males, CON vs. OVER females, and CON vs. OVER males, respectively. Locations of DMRs were determined using Ensemble. DMRs were considered to be located in a promoter region if they were within 5 kb upstream from the start site of a gene.

A comparison of the CON females with the RES females identified 2416 DML and 34 DMRs (Table 2). A lesser amount of DML (1874) and DMRs (48) were identified when comparing the CON females with the OVER females (Table 2). For the comparison of the CON vs. the RES males, 1514 DML and 64 DMRs were identified. A total of 608 DML and 25 DMRs were identified when comparing the CON males with the OVER males (Table 2). The majority of the DMRs identified were located in introns (Figure 4), with these results being similar within the respective sexes.

The offspring from ewes fed 100% (CON), 60% (RES), or 140% (OVER) of NRC requirements for TDN from d 30.2 to d 135 of gestation. DML: Differentially methylated loci; DMRs: Differentially methylated regions.

**Figure 5:**
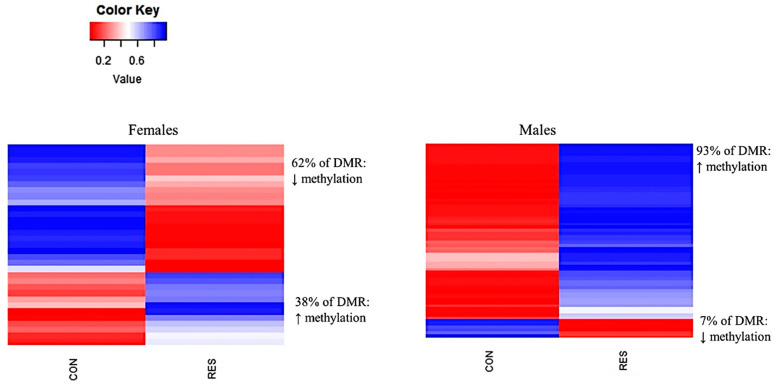
Exposure to nutrient restriction during gestation changes fetal pancreas tissue DNA methylation in a sex-specific manner. Fetuses collected from control- and restricted-fed ewes at d 135 of gestation are referred to as CON and RES, respectively. Blue = hypermethylated; red = hypomethylated. Regions were considered differentially methylated when *p* ≤ 0.001 and ∆ = 0.10.

A smaller percentage of DMRs were identified within a promoter region (promoter region = 5 kb from a transcription start site; 9% for CON vs. RES females and CON vs. OVER females, and 7% for CON vs. RES males; Figure 4). The DMRs that were located in a gene (Intergenic DMRs) were also identified in both the male and female offspring in all three treatment groups (Figure 4). For the DMRs identified in the RES females, 62% were hypomethylated and 38% were hypermethylated when compared with the CON females (*p* = 0.001; Figure 5).

**Figure 6:**
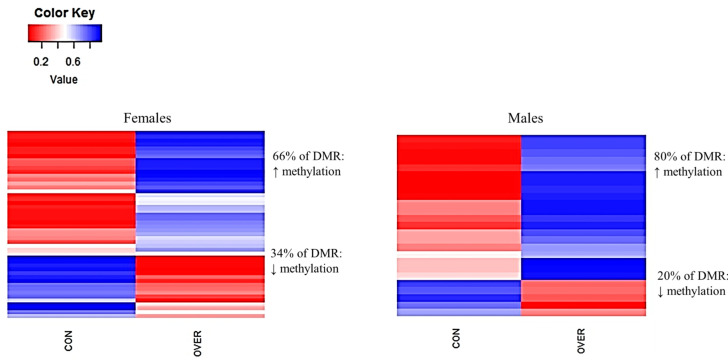
Maternal over-nutrition during gestation increases DNA methylation in male and female offspring. Fetuses collected from control- and over-fed ewes at d 135 of gestation are referred to as CON and OVER, respectively. Blue = hypermethylated; red = hypomethylated. Regions were considered differentially methylated when *p* ≤ 0.001 and ∆ = 0.10.

In comparing the DMRs of the RES male offspring with the CON males, 93% were hypermethylated and 7% were hypomethylated (*p* = 0.001; Figure 5). In the OVER females, 66% of the DMRs were hypermethylated, while 34% of DMRs were hypomethylated compared with CON (*p* = 0.001; Figure 6).

Similarly, 80% of the DMRs identified in the OVER males were hypermethylated compared with the CON male fetuses (*p* = 0.001; Figure 6). No common DMRs were identified between the CON vs. RES and CON vs. OVER females (Tables S1 and S2). Only one common region was identified (intron 3 of FBXL (F-Box and Leucine Rich Repeat Protein)-2 gene) in the RES and OVER males when compared with the CON males (Tables S3 and S4). Gene ontology analysis was performed for DMR genes that were found to be within a promoter region or intragenic for cellular component and molecular function. Both the molecular function as well as the cellular component classifications varied greatly within and between the treatment groups. The locations of factors in a cell (e.g., cellular component) included the cellular membrane, ribosome, mitochondria, and the nucleus. All the treatment comparisons had several genes responsible for regulating aspects of cell structure as well as function. Two protein coding genes involved in the mitogen activated protein kinase (MAPK) signaling pathway, CACNG (Calcium Voltage-Gated Channel Auxiliary Subunit Gamma)-5 and Mitogen-Activated Protein Kinase Kinase Kinase (MAP3K)-15, were hypomethylated in the RES females when compared with the CON females (Table 3 and Table S1). In the OVER females, *Claudin (CLDN)-15* was found to be hypomethylated, whereas *NSF Attachment Protein Alpha (NAPA)* exhibited hypermethylation when compared with the CON females (Table 4 and Table S3). Both of these factors are involved in regulating aspects of cellular structure (Table 4). 5-Oxoprolinase, ATP-Hydrolyzing (OPLAH), and SEC23 Homolog B, COPII Coat Complex Component (SEC23B), which are involved in ATP binding and zinc ion binding, respectively, were hypomethylated in the RES males (Table 5).

In the OVER males, the hypermethylation of structural proteins genes MAP7 Domain Containing (MAP7D)-1 and Envoplakin (EVPL) was identified (Table 5 and Table S4). Several of the genes that were differentially methylated in the CON vs. OVER females are involved in regulating transcription and other epigenetic modifications (SET Domain Containing D3 (SETD3), Homeobox C (HOXC)-13, Myomesin (MYOM)-3, and Paired Box (PAX)-5). Likewise, in the CON vs. RES and CON vs. OVER males (Table 6), genes were identified with similar transcriptional regulatory functions. However, the differentially methylated genes were different than those identified in the female offspring.

## 4. Discussion

Pancreas tissue development begins during early gestation, with the production of insulin from β-cells observed as early as d 29 of gestation in sheep [3]. Therefore, the pancreas, similar to many other organs of the developing fetus, is susceptible to the effects of fetal programming. However, the mechanisms by which these changes occur are poorly understood. In the current study, we determined that maternal restricted- and over-nutrition during gestation resulted in changes to the islet size, islet number, β-cell proliferation, and DNA methylation. These data demonstrate that maternal under- and over-nutrition during gestation (1) alters offspring pancreatic development and (2) facilitates changes to offspring pancreas growth and function postnatally.

The expansion of β-cell mass occurs during late gestation [23], which is likely why the majority of the differences we observed were at the d 135 time point. We identified a greater islet size and number in the RES and OVER offspring, despite similar nuclei numbers. Consequently, the observed increase in islet size is likely due to cellular hypertrophy. Cellular hypertrophy has been reported in the pancreatic islets of neonates from diabetic mothers [24] as well as during the early stages of β-cell dysfunction [25]. It was expected that the RES fetuses would have a smaller pancreas as well as a proportionate reduction in islet number and islet size, as this has been observed in other fetal programming models (e.g., nutrient restriction and placental insufficiency) [18,26,27]. It is important to note that the pancreas of the RES fetuses were smaller at d 135 of gestation. However, when adjusted for animal body weight, the difference was no longer observed. Therefore, it is plausible that the increase in cell size may have been an adaptive response to account for the differences in pancreas mass.

Beta-cell expansion also occurs early on in postnatal life in mice, rats, and humans [28]. During this time, the pancreas undergoes a remodeling event where there is increased cellular apoptosis and proliferation. In young lambs, islet remodeling occurs during the first ten days of life [29]. Therefore, the reduction in cellular proliferation observed in OVER animals at birth could impact early postnatal pancreatic remodeling and predispose the offspring for altered insulin production later in life. Research needs to be conducted in sheep to better understand early postnatal pancreas remodeling events and how maternal diet during gestation may affect this process.

Maternal diet has also been found to affect the production of hormones by the islets of offspring. For example, Ford et al. ([10]) determined that over-nutrition ewes, 60 days prior to breeding and throughout pregnancy, resulted in increased circulating insulin and glucose concentrations in the dam as well as a greater insulin positive area in the fetal pancreas tissue at the fetus at d 75 of gestation. This is similar to our findings in the OVER fetuses at d 135 of gestation. In the present study, the increase in the insulin positive area was coupled with a reduction in the glucagon positive area. These differences are likely due to the OVER fetuses responding to the increased nutrient availability that has resulted from the over-nutrition. In the RES animals, a tendency for an increased insulin positive area was observed, coupled with an increase in the somatostatin positive area. Keomanivong et al. (2016; [27]) reported a reduction in the insulin positive area in RES fetuses at d 130 of gestation. However, the dams in thatstudy began diets later during gestation (at d 50) and were nulliparous. Typically, a greater effect of fetal programming is exhibited in nulliparous females [30]. All the ewes in the present study were multiparous, which could explain the differences observed between these two studies. Somatostatin is responsible for inhibiting the release of insulin and glucagon, as well as digestive enzymes from the exocrine pancreas [31]. The increase in the somatostatin positive area in the RES offspring at d 135 could be to counteract the increase in insulin production in the pancreas tissue of these animals.

The histological differences observed in the RES and OVER fetuses at d 135 of gestation were not maintained at birth. However, these lambs had just begun to consume milk and animal sampling was not timed based on meal consumption. Therefore, this could have introduced variability that contributed to the lack of differences observed. If we had followed these animals until a later time point, it is likely that differences in insulin production in the pancreas tissue would have been observed. For example, we previously reported that 1 to 3-month-old lambs born to over-fed ewes had increased circulating insulin concentrations and lambs born to restricted-fed ewes exhibited an increased insulin:glucose ratio when an intravenous glucose tolerance test was performed [32]. Overall, additional studies are needed that follow these offspring long-term to determine if these differences are maintained and their impact on postnatal pancreas growth and development.

To better understand how maternal nutrition can alter the development of the offspring’s pancreas tissue, DNA methylation patterns were evaluated. DNA methylation is responsible for regulating key processes including cellular differentiation and gene expression [12]. It has been demonstrated that fetal programming during pregnancy can affect the DNA methylation patterns in the tissue of the offspring [15,33,34]. For example, Park et al. (2008; [34]) determined that the DNA methylation of the *PDX1* promoter in pancreas tissues was increased in IUGR rats, leading to gene silencing. Additionally, it has been suggested that alternations to DNA methylation and other key epigenetic mechanisms may lead to the development of type II diabetes later in life [35]. In general, our data agree with our hypotheses and the aforementioned findings. However, the dietary specific changes to the DNA methylation patterns we observed is different than originally anticipated. We expected a reduction in DNA methylation in both the male and female RES offspring, as protein restriction has been indicated to reduce DNA methylation in rat fetal programming models [36]. However, only the female offspring responded in this manner. These sexual dimorphic effects also appear to be diet-specific because in both the male and female OVER animals, an increased pancreas DNA methylation was observed. To date, many studies have demonstrated the sex-specific effects of fetal programming on the growth, development, and organogenesis of the offspring [6]. Our data demonstrate that these differences occur at the epigenetic level as well. In the context of the pancreas tissue, our findings are similar to those of Hall et al. (2014; [37]), who demonstrated that human male and female diabetes patients exhibited differences in DNA methylation patterns in the islets of the pancreas tissue. It has been postulated that this is due to the differences in pancreatic function that exist between males and females [37].

In the present study, changes to methylation patterns were observed in the promoter regions, introns, and exons of a multitude of different genes. Many of these genes are involved in regulating the transport of molecules into/within the cell, cell signaling, and gene expression. It is likely that this could be mediating some of the changes in the pancreas histology that we observed in the offspring at d 135 of gestation; however, without functional studies, these conclusions would be largely speculative. Instead, it is important to note the common “themes” in certain families of factors that we identified in this study that warrant further investigation and are similar to the findings of others. For example, members of the Transmembrane (*TMEM*) family are proteins that are embedded in the membrane of the cell and its organelles. These proteins transport molecules across the membranes and are involved in inflammation, cell signaling, and oncogenesis [38,39]. The hypomethylation of *TMEM55A* introns was observed in RES females. Zhu et al. (2019; [40]) determined that gestational diabetes (GDM) in a murine model increased the gene body DNA methylation of *TMEM117, 134,* and *151b* in the pancreases tissue of offspring [40]. While the amount of DNA methylation is different, the identification of differential methylation in the two different fetal programming models is notable. The hypermethylation of specific regions has been observed in the present study and in others. For example, GDM offspring exhibited hypermethylation in the *HOXA3* and *A5* gene regions [40]. In our study, *HOXC13* and *HOXD3* also exhibited hypermethylation. Additionally, the promoter region of *AGAP2* has also been found to be hypermethylated in GDM-born offspring [40], which is similar to our findings in RES male offspring [36]. Given the key role of *HOX* genes in cell differentiation, function, and cancer [41] as well as *AGAP2* as a proto-oncogene [42], additional work needs to be conducted to determine if changes in the expression of these genes could alter the histology and function of the pancreas tissue.

Changes to intragenic DNA methylation were also observed in the present study for histone methyltransferases (*KMT2C* and *SETD3*) and histone acetyl transferases (*KAT6A*) in the offspring. This suggests the potential involvement of other epigenetic modifications. This is not surprising as histone modifications will affect the DNA methylation patterns of the promoters involved in pancreas development [43]. Therefore, these factors need to be evaluated at both the epigenetic and protein level in future studies to better understand this relationship.

## 5. Conclusions

From the present study, we determined that maternal under- and over-nutrition during gestation affects the development of the pancreas tissue causing changes to islet size, islet number, and beta cell proliferation. Alterations to the pancreas development at these critical stages early in life could contribute to impaired pancreas function during adulthood. We determined that maternal under- and over-nutrition during gestation affects the DNA methylation patterns of the pancreatic tissue of the offspring offspring’s pancreas tissue in a sex-dependent and diet-specific manner. Additional studies need to be conducted to determine the functional implications of the changes to the DNA methylation patterns that we observed as well as to evaluate the long-term effects of maternal diet on the DNA methylation patterns of the offspring.

## Figures and Tables

**Table 1:** RRBS Sequencing Information Summary.

	Treatment		
	CON	RES	OVER
Total # Reads ^1^			
Males	4,999,063	5,261,852	4,830,722
Females	7,810,231	4,225,227	6,023,562
			
# Reads Post Trimming ^2^			
Males	4,903,981	4,174,833	3,310,469
Females	6,844,615	4,252,496	5,386,296
% Mapping ^3^			
Males	24	21	26
Females	44	41	43

^1^ Total number of raw reads obtained from sequencing ^2^ Total number of reads available for mapping after trimming and removal of short or low-quality reads ^3^ Mapping efficiency of sequences mapped to the *Ovis aries* reference annotation (Oar_V.3.1) using Bismark.

**Table 2:** Offspring pancreas tissue DML and DMRs.

Treatment Comparison	Sex	DML	DMR
CON vs. RES	Female	2416	34
CON vs. RES	Male	1514	64
CON vs. OVER	Female	1874	48
CON vs. OVER	Male	608	25

**Table 3:** Gene Ontology for DMRs identified in CON vs. RES Females.

Gene Name	Molecular Function	Cellular Component
*EFCAB6*	GO:0005509: calcium ion binding	N/A
*CACNG5*	GO:0005245: voltage-gated calcium channel activity; GO:0016247: channel regulator activity	GO:0032281: AMPA glutamate receptor complex
*HDLBP*	GO:0003723: RNA binding	N/A
*KBTBD12*	N/A	GO:0031463: Cul3-RING ubiquitin ligase complex
*MAP3K15*	GO:0004709: MAP kinase kinase kinase activity; GO:0005524: ATP binding	GO:0005622: Intracellular
*SLC10A5*	GO:0008508: bile acid: sodium symporter activity	GO:0005887: Integral component of plasma membrane
*TXNDC11*	GO:0003756: protein disulfide isomerase activity	GO:0005623: Cell; GO:0005783: endoplasmic reticulum; GO:0005789: Endoplasmic reticulum membrane; GO:0016021: Integral component of membrane
*TCN1*	GO:0031419: cobalamin binding	GO:0005615: Extracellular space
*TMEM55A*	GO:0034597: phosphatidylinositol-4,5-bisphosphate 4-phosphatase activity	GO:0005765: Lysosomal membrane; GO:0016021: integral component of membrane; GO:0031902: late endosome membrane
*WDR48*	N/A	GO:0005634: Nucleus; GO:0005764: lysosome; GO:0005770: Late endosome

The DMRs identified were analyzed using DAVID v 6.8 [21,22]. *EF-Hand CalcoumBinding Domain 6 (EFCAB6), Calcium Voltage Channel Auxiliary Subunit Gamma 5 (CACNG5), Vigilin (HDLBP), Kelch Repeat, and BTB Domain Containing 12 (KBTBD12), Mitogen-Activated Protein Kinase Kinase Kinase 15 (MAP3K15), Solute Carrier Family 10 Member 5 (SLC10A5), Thioredoxin Domain Containing 11 (TXNDC11), Transcobalamin 1 (TCN1), Transmembrane Protein 55A (TMEM55A), WD Repeat Domain 48 (WDR48)*.

**Table 4:** Gene Ontology for DMRs identified in CON vs.OVER Females.

Gene Name	Molecular Function	Cellular Component
*HMGCL*	GO:0000287: magnesium ion binding; GO:0004419: hydroxymethylglutaryl-CoA lyase activity; GO:0005102: receptor binding; GO:0030145: manganese ion binding; GO:0042803: protein homodimerization activity; GO:0046872: metal ion binding	GO:0005743: mitochondrial inner membrane; GO:0005759: mitochondrial matrix; GO:0005777: peroxisome
*NAPA*	GO:0005483: soluble NSF attachment protein activity; GO:0019905: syntaxin binding	GO:0005774: vacuolar membrane; GO:0031201: SNARE complex; GO:0043209: myelin sheath; GO:0070044: synaptobrevin 2-SNAP-25-syntaxin-1a complex; GO:0070062: extracellular exosome
*SETD3*	GO:0003713: transcription coactivator activity; GO:0042800: histone methyltransferase activity (H3-K4 specific); GO:0046975: histone methyltransferase activity (H3-K36 specific)	GO:0000790: nuclear chromatin
*TNFRSF19*	GO:0004872: receptor activity	GO:0005886: plasma membrane; GO:0016021: integral component of membrane
*CHMP4C*	N/A	GO:0070062: extracellular exosome; GO:0090543: Flemming body
*CLDN15*	GO:0005198: structural molecule activity	GO:0005886: plasma membrane; GO:0005923: bicellular tight junction; GO:0016021: integral component of membrane; GO:0016328: lateral plasma membrane
*HOXC13*	GO:0001077: transcriptional activator activity; RNA polymerase II core promoter proximal region sequence-specific binding; GO:0003682: chromatin binding; GO:004356: sequence-specific DNA binding	GO:0005634: nucleus
*KMT2C*	GO:0008270: zinc ion binding; GO:0042800: histone methyltransferase activity (H3-K4 specific); GO:0044822: poly(A) RNA binding	GO:0044666: MLL3/4 complex
*LPGAT1*	N/A	GO:0005737: cytoplasm; GO:0016021: integral component of membrane
*MYOM3*	GO:0016746: transferase activity, transferring acyl groups	GO:0031430: M band
*MYH11*	GO:0003774: motor activity; GO:0005524: ATP binding; GO:0008307: structural constituent of muscle	GO:0016459: myosin complex; GO:0070062: extracellular exosome
*PAX5*	GO:0000978: RNA polymerase II core promoter proximal region sequence-specific DNA binding; GO:0001077: transcriptional activator activity, RNA polymerase II core promoter proximal region sequence-specific binding	GO:0005634: nucleus

DMRs identified were analyzed using DAVID v 6.8 [21,22]. *3-hydroxymethyl-3-methylglutaryl-CoA lyase (HMGCL), NSF attachment protein alpha (NAPA), SET domain containing 3 (SETD3), TNF receptor superfamily member 19 (TNFRSF19), charged multivesicular body protein 4C (CHMP4C), claudin 15 (CLDN15), homeobox C13 (HOXC13), lysine methyltransferase 2C (KMT2C), lysophosphatidylglycerol acyltransferase 1 (LPGAT1), myomesin 3 (MYOM3), myosin heavy chain 11 (MYH11), paired box 5 (PAX5)*.

**Table 5:** Gene ontology DMRs identified in CON vs. RES males.

Gene Name	Molecular Function	Cellular Component
*OPLAH*	GO:0005524: ATP binding; GO:0016787: hydrolase activity; GO:0017168: 5-oxoprolinase (ATP-hydrolyzing) activity	GO:0005829: cytosol
*AGAP2*	GO:0005096: GTPase activator activity; GO:0005525: GTP binding	GO:0005622: intracellular
*EVL*	N/A	GO:0005737: cytoplasm; GO:0005856: cytoskeleton; GO:0005925: focal adhesion; GO:0016020: membrane; GO:0030027: lamellipodium
*FBXL2*	GO:0004842: ubiquitin-protein transferase activity; GO:0005516~calmodulin binding, GO:0019903~protein phosphatase binding, GO:0036312~phosphatidylinositol 3-kinase regulatory subunit binding,	GO:0016020: membrane; GO:0019005: SCF ubiquitin ligase complex
*PPARGC1B*	GO:0000166: nucleotide binding; GO:0001104:RNA polymerase II transcription cofactor activity, GO:0003676: nucleic acid binding; GO:0008134:transcription factor binding; GO:0030374:ligand-dependent nuclear receptor transcription coactivator activity; GO:0030546: receptor activator activity	GO:0005634: nucleus; GO:0005739: mitochondrion; GO:0016592: mediator complex
*SEC23B*	GO:0008270: zinc ion binding	GO:0000139: Golgi membrane; GO:0005783: endoplasmic reticulum; GO:0005789: endoplasmic reticulum membrane; GO:0005794: Golgi apparatus; GO:0030127: COPII vesicle coat; GO:0033116: endoplasmic reticulum-Golgi intermediate compartment membrane; GO:0048471: perinuclear region of cytoplasm
*XPC*	GO:0003684: damaged DNA binding; GO:0003697: single-stranded DNA binding	GO:0000111: nucleotide-excision repair factor 2 complex; GO:0005730: nucleolus; GO:0005737: cytoplasm; GO:0005886: plasma membrane; GO:0070062: extracellular exosome; GO:0071942: XPC complex
*CNTNAP5*	N/A	GO:0016021:integral component of membrane
*DAGLB*	GO:0016787: hydrolase activity	GO:0005765: lysosomal membrane; GO:0005886: plasma membrane; GO:0016021: integral component of membrane
*DUSP16*	GO:0004725: protein tyrosine phosphatase activity; GO:0017017: MAP kinase tyrosine/serine/threonine phosphatase activity	GO:0005654: nucleoplasm; GO:0005737: cytoplasm
*HOXD3*	GO:0003700: transcription factor activity, sequence-specific DNA binding; GO:0043565: sequence-specific DNA binding	GO:0005654: nucleoplasm; GO:0016235: aggresome
*IGSF3*	N/A	GO:0005886: plasma membrane; GO:0009986: cell surface; GO:0016021: integral component of membrane
*ITGB2*	GO:0001948: glycoprotein binding; GO:0005515: protein binding; GO:0019901: protein kinase binding; GO:0030369: ICAM-3 receptor activity; GO:0046872: metal ion binding; GO:0050839: cell adhesion molecule binding	GO:0009986: cell surface; GO:0016020: membrane; GO:0034687: integrin alphaL-beta2 complex; GO:0070062: extracellular exosome
*ITIH4*	GO:0004867: serine-type endopeptidase inhibitor activity	GO:0005576: extracellular region; GO:0005737: cytoplasm; GO:0005886: plasma membrane; GO:0070062: extracellular exosome; GO:0072562: blood microparticle
*KAT6A*	GO:0003677: DNA binding; GO:0003713: transcription coactivator activity; GO:0004402: histone acetyltransferase activity; GO:0008270: zinc ion binding	GO:0000786: nucleosome; GO:0005794: Golgi apparatus; GO:0016605: PML body; GO:0070776: MOZ/MORF histone acetyltransferase complex
*PALLD*	N/A	GO:0005634: nucleus; GO:0005739: mitochondrion; GO:0005884: actin filament; GO:0005886: plasma membrane; GO:0005925: focal adhesion
*PLXNA2*	GO:0017154: semaphorin receptor activity	GO:0002116: semaphorin receptor complex; GO:0005887: integral component of plasma membrane
*SEMA3B*	GO:0030215: semaphorin receptor binding; GO:0038191: neuropilin binding; GO:0045499: chemorepellent activity	GO:0005615: extracellular space
*SEMA5A*	GO:0030215: semaphorin receptor binding; GO:0045499: chemorepellent activity	GO:0016021: integral component of membrane; GO:0070062: extracellular exosome
*TACC2*	N/A	GO:0005730: nucleolus; GO:0005737: cytoplasm; GO:0015630: microtubule cytoskeleton
*ZDHHC5*	GO:0008270: zinc ion binding; GO:0016409: palmitoyltransferase activity; GO:0019706: protein-cysteine S-palmitoyltransferase activity	GO:0005737: cytoplasm; GO:0005886: plasma membrane; GO:0016021: integral component of membrane; GO:0030425: dendrite
*ZNF469*	N/A	GO:0003676: nucleic acid binding; GO:0046872: metal ion binding

The DMRs identified were analyzed using DAVID v 6.8 [21,22]. *5-oxoprolinase (ATP-hydrolyzing (OPLAH), ArfGAP with GTPase domain, ankyrin repeat and PH domain 2(AGAP2), Enah/Vasp-like (EVL), F-box and leucine rich repeat protein 2 (FBXL2), PPARG coactivator 1 beta (PPARGC1B), Sec23 homolog B, coat complex II component (SEC23B), XPC complex subunit, DNA damage recognition and repair factor (XPC), contactin associated protein like 5 (CNTNAP5), diacylglycerol lipase beta (DAGLB), dual specificity phosphatase 16 (DUSP16), homeobox D3 (HOXD3), immunoglobulin superfamily member 3 (IGSF3), integrin subunit beta 2 (ITGB2), inter-alpha-trypsin inhibitor heavy chain family member 4 (ITIH4), lysine acetyltransferase 6A (KAT6A), palladin, cytoskeletal associated protein (PALLD), plexin A2 (PLXNA2), semaphorin 3B (SEMA3B), semaphorin 5A (SEMA5A), transforming acidic coiled-coil containing protein 2 (TACC2), zinc finger DHHC-type containing 5 (ZDHHC5), zinc finger protein 469 (ZNF469)*.

**Table 6:** Gene ontology DMRs identified in CON vs. OVER males.

Gene Name	Molecular Function	Cellular Component
*FBXL2*	GO:0004842: ubiquitin-protein transferase activity; GO:0005516: calmodulin binding; GO:0019903: protein phosphatase binding; GO:0036312: phosphatidylinositol 3-kinase regulatory subunit binding	GO:0016020: membrane; GO:0019005: SCF ubiquitin ligase complex
*MAP7D1*	GO:0005198: structural molecule activity	GO:0015630: microtubule cytoskeleton
*B3GNT8*	GO:0008378: galactosyltransferase activity; GO:0016262: protein N-acetylglucosaminyltransferase activity	GO:0000139: Golgi membrane; GO:0016021: integral component of membrane; GO:0070062: extracellular exosome
*ABLIM2*	GO:0008270: zinc ion binding	GO:0015629: actin cytoskeleton
*EVPL*	GO:0005198: structural molecule activity	GO:0001533: cornified envelope; GO:0005737: cytoplasm; GO:0045111: intermediate filament cytoskeleton; GO:0070062: extracellular exosome
*HAGH*	GO:0004416: hydroxyacylglutathione hydrolase activity; GO:0046872: metal ion binding	GO:0005759: mitochondrial matrix; GO:0070062: extracellular exosome
*ING5*	GO:0003682: chromatin binding; GO:0008270: zinc ion binding	GO:0070776: MOZ/MORF histone acetyltransferase complex
*OPRL1*	GO:0001626: nociceptin receptor activity; GO:0042923: neuropeptide binding	GO:0005887: integral component of plasma membrane; GO:0043005: neuron projection

The DMRs identified were analyzed using DAVID v 6.8 [21,22]. *F-box and leucine rich repeat protein 2 (FBXL2), MAP7 domain containing 1 (MAP7D1), UDP-GlcNAc:betaGal beta-1,3-N-acetylglucosaminyltransferase8(B3GNT8), actin binding LIM protein family member 2 (ABLIM2), Envoplakin (EVPL), hydroxyacylglutathione hydrolase (HAGH), inhibitor of growth family member 5 (ING5), opioid related nociceptin receptor 1 (OPRL1)*.

## Data Availability

Data are in the process of being posted to a repository and all information will be made available prior to publication.

## Supplementary Materials

The following are available online at https://www.mdpi.com/article/10.3390/ani11092531/s1, Table S1: DMRs CON v RES Female, Table S2: DMRs CON v RES Male, Table S3: DMRs CON v OVER Females, Table S4: DMRs CON v OVER males.
