# Supplementary material for: Maternal Under- and Over-Nutrition during Gestation Causes Islet Hypertrophy and Sex-Specific Changes to Pancreas DNA Methylation in Fetal Sheep
Source: Animals (Basel). 2021 Aug 28;11(9):2531. doi: 10.3390/ani11092531 (PMC8466738; doi:10.3390/ani11092531)
Supplement: Supplementary file 1 [file animals-11-02531-s001.zip › Table S1.pdf]

Table S1: DMR CON vs. RES Females

Position	Length	nCG	CON Mean Methyl	RES Mean Methyl	Description
1:464715-464771	57	5	0.76	0.04	2.4 KB away from *HDLBP* gene
2:239653860-239653940	81	6	0.91	0.28	1.066 KB away from *AIM1L*
16:68991039-68991095	57	4	0.06	0.72	2.769 KB away from ENSOART00000029041 and 2.728KB away from ENSOART00000006290
3:218500576-218500627	52	5	0.84	0.23	Intron of *EFCAB6* AND SNPS
6:114757255-114757320	66	5	0.19	0.74	Intron of ENSOART00000012888
7:97968446-97968507	62	7	0.56	0.03	Intron of ENSOART00000003646
9:86033664-86033753	90	13	0.70	0.28	Intron of *TMEM55A*
9:93437818-93437878	61	11	0.89	0.34	Intron of *SLC10A5*
11:61882803-61882874	72	5	0.16	0.72	Intron of *CACNG5*
11:61883532-61883591	60	9	0.17	0.62	Exon of *CACNG5*
15:80037597-80037659	63	10	0.22	0.83	*TCN1* and LincRNA and ENSART00000028887
15:54729901-54729957	57	4	0.92	0.05	Intron of *MY07A*
16:71007561-71007626	66	4	0.89	0.08	Intron of ENSOART00000029051 and ENSOART00000017567 and 17558
19:12309493-12309543	51	5	0.32	0.74	Intron of *WDR48*
19:59382698-59384364	1667	8	0.65	0.28	Intron of *KBTBD12*
20:46494357-46494436	80	7	0.77	0.34	Intron of ENSOART00000018791
24:517119-517175	57	5	0.71	0.25	Exons of ENSOART00000014492 and ENSOART00000014379
24:10372469-10372551	83	7	0.87	0.05	Exon of *TXNDC11*
X:16844758-16844835	78	13	0.10	0.67	Exon of *MAP3K15*
1:1650602-1650669	68	11	0.84	0.25	Intron
4:118512424-118512488	65	7	0.82	0.39	Intron
5:37271183-37271264	82	9	0.08	0.53	Intron
10:10836737-10836818	82	13	0.26	0.79	Intron
11:52284621-52284678	58	4	0.91	0.27	Intron
14:17657754-17657829	76	5	0.92	0.09	Intron

20:14551751-14551812	62	7	0.20	0.58	Intron
20:50995235-50995299	65	6	0.95	0.12	Intron
20:49813946-49814021	76	17	0.38	0.90	Intron
23:59841258-59841324	67	5	0.93	0.07	Intron
25:39228278-39228365	88	4	0.04	0.89	Intron

Position[1]: Defines the chromosome and specific loci where the DMR is located; Length[2]: The length in base pairs of the DMR; nCG[3]: number of CpG dinucleotides within the region of interest; CON and RES mean methyl[4]: the numerical representation of DNA methylation at the CpG dinucleotides in this region for a given treatment group. Determined by DSS with 0 being the minimum and 1 being the maximum.
