# Supplementary material for: Maternal Under- and Over-Nutrition during Gestation Causes Islet Hypertrophy and Sex-Specific Changes to Pancreas DNA Methylation in Fetal Sheep
Source: Animals (Basel). 2021 Aug 28;11(9):2531. doi: 10.3390/ani11092531 (PMC8466738; doi:10.3390/ani11092531)
Supplement: Supplementary file 1 [file animals-11-02531-s001.zip › Table S2.pdf]

Table S2. DMR CON vs. RES Males

Position	length	nCG	CON Mean Methyl	RES Mean Methyl	Description
3:202159208-202159267	60	6	0.07	0.85	3.065 KB away from *DUSP16*
5:58665088-58665159	72	5	0.38	0.89	4.905 kb away from *PPARGC1B*
11:54422768-54422855	88	7	0.06	0.78	1.296 KB away from *QRICH2*
15:78117923-78118004	82	4	0.07	0.89	583 bp away from *ZDHHC5*
1:93136505-93136556	52	4	0.27	0.91	*IGSF3*; Exon 2 of 3
1:262873778-262873831	54	5	0.08	0.53	*ITGB2*; Exon 10 of 17
2:189246693-189246840	148	4	0.09	0.92	*CNTNAP5*; intron 12 of 22
2:132848318-132848374	57	7	0.17	0.87	*HOXD3*; Intron 1 of 3
2:239803845-239803908	64	7	0.04	0.60	*CNKSR1*; Intron 1of 21
2:110899932-110899989	58	23	0.16	0.58	*PALLD*; Intron 11 of 22
3:161641254-161641314	61	10	0.08	0.91	*AGAP2*; exon 22 and exon 18
5:49331321-49331393	73	4	0.15	0.69	*PCDHA5*; INTRON
6:113430863-113430929	67	6	0.33	0.92	*SORCS2*; Exon 1 of 14
8:73546567-73546618	52	5	0.08	0.76	*7C3H12D*; Intron 3 of 5
9:13718226-13718282	57	9	0.78	0.15	*OPLAH*; Intron 4 of 24
12:73332410-73332460	51	5	0.38	0.89	*PLXNA2*; INTRON
13:37691865-37691947	83	5	0.90	0.07	*SEC23B*; Exon 5 of 20
14:13211805-13211903	99	8	0.84	0.05	*ZNF469*; Exon 11 of 11
16:63840143-63840242	100	9	0.08	0.91	*SEMA5A*; Exon 5 of 24
17:63069786-63069849	64	5	0.06	0.71	*TRPL4*; EXON/INTRON
18:63666165-63666245	81	6	0.10	0.93	*EVL*; intron 1 of 15
19:50037484-50037584	101	10	0.05	0.85	*SEMA3B*; Exon 19 of 19
19:48041293-48041384	92	7	0.08	0.87	*ITIH4*; Exon 4 of 23
19:57887666-57887722	57	4	0.09	0.81	*XPC*; Exon 4 of 21
19:7525598-7525651	54	5	0.37	0.88	*FBXL2*; Intron 3 of 17
20:50389049-50389109	61	6	0.21	0.92	*GMDS*; Intron l 7 of 8
22:40964876-40964966	91	8	0.08	0.90	*TACC2* INTRON 7/11 AND 6/21
24:38193510-38193603	94	9	0.88	0.07	*DAGLB*; INTRON 2 of 13 AND ENSOART INTRON
26:35359136-35359190	55	6	0.09	0.68	*KAT6A*; Intron 7 of 18
1:112851565-112851620	56	4	0.15	0.86	Intron
3:77936730-77936793	64	4	0.08	0.90	Intron
3:1301392-1301454	63	7	0.11	0.89	Intron
4:5110018-5110069	52	4	0.05	0.86	Intron
6:114796343-114796395	53	4	0.07	0.90	Intron
8:87098382-87098435	54	5	0.21	0.83	Intron
9:16404705-16404788	84	5	0.08	0.92	Intron
9:27465758-27465846	89	7	0.05	0.87	Intron

10:83894191-83894264	74	6	0.07	0.89	Intron
10:85913053-85913133	81	9	0.08	0.82	Intron
10:31189762-31189825	64	6	0.09	0.76	Intron
14:13721605-13721704	100	4	0.08	0.91	Intron
14:58805301-58805353	53	4	0.04	0.66	Intron
15:25770093-25770168	76	7	0.07	0.87	Intron
16:69499064-69499121	58	4	0.05	0.89	Intron
18:26740266-26740322	57	4	0.06	0.70	Intron
20:19475895-19475947	53	4	0.05	0.68	Intron
20:49611584-49611635	52	5	0.19	0.77	Intron
20:10592049-10592100	52	7	0.07	0.52	Intron
20:43916067-43916121	55	6	0.82	0.07	Intron
20:16182376-16182444	69	4	0.92	0.17	Intron
23:46399514-46399590	77	5	0.08	0.91	Intron
26:5357940-5357993	54	4	0.07	0.91	Intron
26:1135785-1135837	53	5	0.24	0.93	Intron

Position[1]: Defines the chromosome and specific loci where the DMR is located ; Length[2]: The length in base pairs of the DMR; nCG[3]: number of CpG dinucleotides within the region of interest; CON and RES mean methyl[4]: the numerical representation of DNA methylation at the CpG dinucleotides in this region for a given treatment group. Determined by DSS with 0 being the minimum and 1 being the maximum.
