# Supplementary material for: Maternal Under- and Over-Nutrition during Gestation Causes Islet Hypertrophy and Sex-Specific Changes to Pancreas DNA Methylation in Fetal Sheep
Source: Animals (Basel). 2021 Aug 28;11(9):2531. doi: 10.3390/ani11092531 (PMC8466738; doi:10.3390/ani11092531)
Supplement: Supplementary file 1 [file animals-11-02531-s001.zip › Table S3.pdf]

Table S3: DMR CON vs. OVER Females

Position	length	nCG	CON Mean Methyl	OVER Mean Methyl	Description
12:47934573-47934638	66	9	0.26	0.57	2.108 KB away from ENSOARG00000005173
17:70880056-70880129	74	6	0.77	0.47	1.660 kb away from *SPECC1L* gene
18:63069833-63069885	53	4	0.10	0.70	3.987 kb away from *SETD3*
24:35371763-35371855	93	4	0.92	0.12	69 bp away from *CLDN15*
2:241992534-241992653	120	4	0.05	0.89	*HMGCL*; intron 5 of 8
2:241724810-241724896	87	4	0.08	0.90	*MYOM3*; Exon 21 OF 37
16:70640052-70640167	116	9	0.13	0.95	Intron of ENSOART00000029045 and ENSOART00000017123
9:93331263-93331326	64	7	0.04	0.78	*CHMP4C*; Intron
11:47328059-47328159	101	13	0.09	0.83	ENSOART00000014446, 5', IN EXON
2:51540059-51540115	57	6	0.19	0.89	*PAX5*; Intron 9 of 9
14:53536170-53536285	116	6	0.19	0.89	*NAPA*; Intron 1 of 10
3:2108909-2108970	62	8	0.09	0.75	ENSOART00000027916, ENSOART00000027915, ENSOART00000002464; Intron between exons
3:132428853-132428913	61	5	0.07	0.73	*HOXC13*; Exon 2 of 3
12:69836775-69836834	60	7	0.27	0.80	*LPGAT1*; Intron 5 and 7 of 8
3:13929171-13929238	68	9	0.11	0.57	*TTILL11*; Intron 9 of 9
18:55012746-55012806	61	7	0.42	0.85	*CCDC88C*; Intron 22 of 30
10:34657676-34657785	110	6	0.19	0.60	*TNFRSF19*; Intron 3 of 8
7:98651520-98651596	77	17	0.29	0.68	ENSOART00000028764; Intron between exons
9:14267289-14267341	53	9	0.42	0.66	ENSOART00000000978; Intron 1 of 1 and ENSOART00000028517; Intron 1
21:49900756-49900825	70	6	0.50	0.62	ENSOART00000010113
5:36481130-36481193	64	9	0.41	0.48	ENSOART00000004682: Exon 2 of 6
9:13139916-13139987	72	6	0.63	0.33	ENSOARG00000026493; Exon 3 of 3
4:114008909-114008998	90	5	0.94	0.39	*KMT2C*; Exon 46 of 66
24:14198052-14198116	65	4	0.63	0.06	*MYH11*: Intron 1 of 39
20:17379447-17379517	71	8	0.75	0.16	ENSOART00000009068: Intron 6 of 7

24:38707723-38707788	66	8	0.85	0.06	ENSOARG00000025897 Intron between exons
5:13991975-13992030	56	4	0.92	0.06	ENSOART0000000590
1:261702406-261702518	113	8	0.09	0.89	Intron
14:49468505-49468597	93	5	0.10	0.87	Intron
4:8704730-8704798	69	7	0.16	0.88	Intron
23:58126139-58126200	62	4	0.10	0.79	Intron
14:57707275-57707373	99	6	0.08	0.75	Intron
1:2342450-2342507	58	5	0.22	0.88	Intron
2:247942155-247942223	69	8	0.27	0.91	Intron
X:125632027-125632092	66	6	0.08	0.62	Intron
X:48437124-48437210	87	9	0.10	0.62	Intron
20:14551322-14551374	53	11	0.33	0.83	Intron
19:56304838-56304889	52	11	0.05	0.53	Intron
19:7083095-7083149	55	11	0.12	0.52	Intron
21:41772133-41772198	66	4	0.29	0.65	Intron
11:33238193-33238294	102	9	0.74	0.26	Intron
24:11489681-11489735	55	7	0.76	0.23	Intron
26:1272854-1272914	61	15	0.78	0.21	Intron
22:48534864-48534936	73	6	0.93	0.34	Intron
4:118424199-118424317	119	6	0.89	0.20	Intron
20:50005171-50005240	70	9	0.89	0.07	Intron
5:59075782-59075888	107	5	0.91	0.05	Intron
11:61046301-61046361	61	8	0.94	0.06	Intron

Position[1]: Defines the chromosome and specific loci where the DMR is located; Length[2]: The length in base pairs of the DMR; nCG[3]: number of CpG dinucleotides within the region of interest; CON and OVER mean methyl[4]: the numerical representation of DNA methylation at the CpG dinucleotides in this region for a given treatment group. Determined by DSS with 0 being the minimum and 1 being the maximum.
