# Supplementary material for: Maternal Under- and Over-Nutrition during Gestation Causes Islet Hypertrophy and Sex-Specific Changes to Pancreas DNA Methylation in Fetal Sheep
Source: Animals (Basel). 2021 Aug 28;11(9):2531. doi: 10.3390/ani11092531 (PMC8466738; doi:10.3390/ani11092531)
Supplement: Supplementary file 1 [file animals-11-02531-s001.zip › Table S4.pdf]

Table S4: DMR CON vs. OVER males

Position	length	nCG	CON Mean Methyl	OVER Mean Methyl	Description
14:49606092-49606178	87	5	0.041680835	0.895398813	*B3GNT8*: Exon 1 of 3
11:54652590-54652670	81	8	0.102872291	0.829746134	*EVPL*: Exon 23 of 25; ENSOART00000009201: Exon 7 of 7
24:1371700-1371798	99	13	0.194786155	0.855225276	*HAGH*: Intron 7 of 9
13:53103530-53103618	89	4	0.265741601	0.906829	*OPRL1*: Exon 3 of 3
20:15672573-15672649	77	4	0.266862843	0.901706398	*FRS3*: Exon 6 of 6
19:7525598-7525651	54	5	0.370443544	0.925768552	*FBXL2*: Intron 3 of 17
1:10843416-10843497	82	9	0.330168738	0.825513102	*MAP7D1*: Exon 10 of 17
6:113784956-113785018	63	5	0.804237098	0.228820095	*ABLIM2*: Intron 7 of 22; ENSOART00000012888: Intron 11 of 12
1:127157-127218	62	4	0.769333287	0.066692206	
22:48153604-48153665	62	6	0.042131761	0.893367642	ENSOART00000016253: INTRON 3 of 10
17:72098904-72098961	58	4	0.05009988	0.877824349	ENSOART00000019397: Intron 1 of 2
11:24630235-24630314	80	4	0.141605495	0.891078618	ENSOART00000020789: Intron 8 of 11
17:71050663-71050722	60	7	0.077644127	0.768991574	ENSOART00000015848: Intron 1 of 21
11:8020039-8020100	62	9	0.414990377	0.916130779	ENSOART00000008542: Intron 9 of 10
3:1520643-1520717	75	4	0.473276777	0.665105887	Lincrna ensoart00000027906
2:246671910-246671979	70	4	0.674297834	0.264657039	LINCRNA ENSOARG00000027830; 2 SNPS
6:44244206-44244271	66	6	0.830603671	0.207785274	1 SNP
22:48153604-48153665	62	6	0.042131761	0.893367642	ENSOART00000016253: Intron 3 of 10
17:72098904-72098961	58	4	0.05009988	0.877824349	ENSOART00000019397: Intron 1 of 2
11:24630235-24630314	80	4	0.141605495	0.891078618	ENSOART00000020789: Intron 8 of 11
17:71050663-71050722	60	7	0.077644127	0.768991574	ENSOART00000015848: Intron 1 of 21
11:8020039-8020100	62	9	0.414990377	0.916130779	ENSOART00000008542: Intron 9 of 10
3:1520643-1520717	75	4	0.473276777	0.665105887	5 SNPS; Lincrna ensoart00000027906
2:246671910-246671979	70	4	0.674297834	0.264657039	LINCRNA ENSOARG00000027830; 2 SNPS
6:44244206-44244271	66	6	0.830603671	0.207785274	1 SNP
16:5003410-5003463	54	6	0.087075185	0.833660409	Intron
15:25817118-25817174	57	5	0.069898905	0.895398813	Intron

13:78193221-78193303	83	5	0.07776261	0.79259617	Intron
12:77589529-77589592	64	6	0.126463782	0.742004317	Intron
24:38102767-38102836	70	5	0.875303149	0.188286781	Intron
16:70927301-70927351	51	5	0.376954719	0.953466008	Intron
3:210701775-210701837	63	6	0.329697687	0.762501829	Intron
11:36250281-36250361	81	4	0.260779749	0.680534441	Intron

Position[1]: Defines the chromosome and specific loci where the DMR is located; Length[2]: The length in base pairs of the DMR; nCG[3]: number of CpG dinucleotides within the region of interest; CON and OVER mean methyl[4]: the numerical representation of DNA methylation at the CpG dinucleotides in this region for a given treatment group. Determined by DSS with 0 being the minimum and 1 being the maximum.
